# Suppressed recombination and unique candidate genes in the divergent haplotype encoding *Fhb1*, a major Fusarium head blight resistance locus in wheat

**DOI:** 10.1007/s00122-016-2727-x

**Published:** 2016-05-12

**Authors:** W. Schweiger, B. Steiner, S. Vautrin, T. Nussbaumer, G. Siegwart, M. Zamini, F. Jungreithmeier, V. Gratl, M. Lemmens, K. F. X. Mayer, H. Bérgès, G. Adam, H. Buerstmayr

**Affiliations:** Institute for Biotechnology in Plant Production (IFA-Tulln), BOKU-University of Natural Resources and Life Sciences, Konrad Lorenz Strasse 20, 3430 Tulln, Austria; French Plant Genomic Resource Centre, INRA-CNRGV, Chemin de Borde Rouge, CS 52627, 31326 Castanet Tolosan, France; Plant Genome and Systems Biology, Helmholtz Zentrum München, 85764 Neuherberg, Germany; Division of Computational System Biology, Department of Microbiology and Ecosystem Science, University of Vienna, 1090 Vienna, Austria; Department of Applied Genetics and Cell Biology, BOKU-University of Natural Resources and Life Sciences, Konrad Lorenz Strasse 22, 3430 Tulln, Austria

## Abstract

****Key message**:**

**Fine mapping and sequencing revealed 28 genes in the non-recombining haplotype containing*Fhb1*. Of these, only a GDSL lipase gene shows a pathogen-dependent expression pattern.**

**Abstract:**

*Fhb1* is a prominent Fusarium head blight resistance locus of wheat, which has been successfully introgressed in adapted breeding material, where it confers a significant increase in overall resistance to the causal pathogen *Fusarium graminearum* and the fungal virulence factor and mycotoxin deoxynivalenol. The *Fhb1* region has been resolved for the susceptible wheat reference genotype Chinese Spring, yet the causal gene itself has not been identified in resistant cultivars. Here, we report the establishment of a 1 Mb contig embracing *Fhb1* in the donor line CM-82036. Sequencing revealed that the region of *Fhb1* deviates from the Chinese Spring reference in DNA size and gene content, which explains the repressed recombination at the locus in the performed fine mapping. Differences in genes expression between near-isogenic lines segregating for *Fhb1* challenged with *F. graminearum* or treated with mock were investigated in a time-course experiment by RNA sequencing. Several candidate genes were identified, including a pathogen-responsive GDSL lipase absent in susceptible lines. The sequence of the *Fhb1* region, the resulting list of candidate genes, and near-diagnostic KASP markers for *Fhb1* constitute a valuable resource for breeding and further studies aiming to identify the gene(s) responsible for *F. graminearum* and deoxynivalenol resistance.

**Electronic supplementary material:**

The online version of this article (doi:10.1007/s00122-016-2727-x) contains supplementary material, which is available to authorized users.

## Introduction

One of the most prevalent pathogens to hexaploid wheat (*Triticum aestivum*) is the hemibiotrophic fungus *Fusarium graminearum*. The related disease Fusarium head blight (FHB) leads to severe reduction in grain yield and quality causing globally devastating economic losses. Infected grain may be contaminated with mycotoxins such as deoxynivalenol (DON) and other heat-stable trichothecene type B toxins, which remain in processed food stuffs and feed and constitute a serious threat to food and feet safety (Pestka 2010). The European Union and many other countries have enacted maximum levels for DON in food stuffs (The European Commission 2006) and the United States Food and Drug Administration has issued advisory levels. Conventional agronomical measures to control the disease, such as changed crop rotations, tillage regimes or the use of fungicides, are costly and/or frequently not applicable. Breeding for resistance against FHB in wheat and other small grain cereals is a sustainable and environmentally friendly strategy to achieve durable and cost efficient resistance.

A broad range of resistance sources exist and about 100 quantitative trait loci (QTL) were described to date by genetic mapping in diverse hexaploid wheat germplasm (Buerstmayr et al. 2009). Yet, most are only minor contributors to overall resistance. Among the strongest and most reliable QTL is *Fhb1*, which has been the focus of several studies aiming to fine-map, identify the causal gene and define its biological mode of action. *Fhb1* (syn. *Qfhs.ndsu*-*3BS*) was first described as a strong contributor to type 2 resistance (resistance against spreading of the disease) located on wheat chromosome 3BS by Waldron et al. (1999) in a biparental recombinant-inbred population derived from the highly resistant Chinese spring wheat landrace Sumai-3 using RFLP markers. The QTL was confirmed by Anderson et al. (2001) with additional SSR markers and a second population generated from Sumai-3 derivative ND2603 as the resistance donor and in an independent study by Buerstmayr et al. (2002), who employed a doubled haploid (DH) population with CM-82036, a CYMMIT-derived offspring of Sumai-3 as the resistance donor. Fine-mapping *Fhb1* (Cuthbert et al. 2006; Liu et al. 2006) narrowed the original confidence interval from SSR markers gwm493 and gwm533 (Anderson et al. 2001) to a 1.2 cM interval between sts3B-189 and sts3B-206 (Liu et al. 2006). BAC sequencing of the syntenic region in the susceptible wheat reference cultivar Chinese Spring yielded UMN10 (Liu et al. 2008), a near-diagnostic marker for *Fhb1* that is widely used for marker-assisted selection in wheat. A single recombinant line in the same study further narrowed the interval down to 0.4 cM with sts3B-32 replacing sts3B-206. The sequence of a large genomic contig harboring the susceptible *Fhb1* region in Chinese Spring has been reported (Choulet et al. 2010) spurring comparisons with the sequence in resistant cultivars. An association mapping study of FHB-related traits identified several significant marker associations yet it seemed difficult to reconcile the genetic map with the physical sequence of the reference genotype (Hao et al. 2012).

Type 2 resistance conferred by *Fhb1* is associated with its ability to inactivate DON. *F. graminearum* requires DON to spread from the initial infection site and penetrate further into the rachis and adjacent spikelets (Jansen et al. 2005). Lemmens et al. (2005) showed that the QTL co-localizes with the higher ability to transform DON into the non-toxin DON-3-O-glucoside. To date, several UDP-glucosyltransferases (UGT) capable of effectively inactivating DON have been identified in several species of the *Gramineae* family (Schweiger et al. 2010, 2013). The barley-derived HvUGT13248 when transformed into susceptible wheat is sufficient to confer high level spreading resistance against *F. graminearum* (Li et al. 2015). Yet, no such gene could be associated with *Fhb1*. Also the genomic region harboring the susceptible *Fhb1* allele from Chinese Spring does not contain a small molecule-accepting UGT gene between markers flanking the QTL.

Several proteomic, metabolomic and transcriptomic studies have sought to pinpoint the mechanism underlying *Fhb1* (Walter et al. 2008; Gunnaiah et al. 2012; Kugler et al. 2013; Schweiger et al. 2013; Xiao et al. 2013; Zhuang et al. 2013; Warth et al. 2014; Nussbaumer et al. 2015; Hofstad et al. 2016). A combined proteomic and metabolomic study by Gunnaiah et al. (2012) suggested that the higher accumulation of phenylpropanoids leads to the resistance effect observed in near-isogenic lines (NILs) harboring the QTL. Using RNA-seq data, Xiao et al. (2013) observed changed jasmonic acid signaling in a deletion mutant of resistant cv. Wangshuibai and suggested this to be as a possible mechanism for the resistance mediated by the QTL. The to date only study that associated transcript abundances to genetically mapped positions stems from a small eQTL study by Zhuang et al. (2013), who suggested that a pectin methyl esterase inhibitor gene mapping into the confidence interval, which is down-regulated in susceptible lines, could be the causal gene. Only the most recent large-scale RNA-seq studies (Nussbaumer et al. 2015; Hofstad et al. 2016) include whole genome mapping data based on the recent release of the full wheat gene models of Chinese Spring (Mayer et al. 2014). Both studies using unrelated pairs of NILs presented viable candidate genes mapped to the *Fhb1* interval that shows stress-dependent and QTL-associated expression, which do show some overlaps to previous transcriptomics studies. Yet, despite the seemingly completeness of the reference genome, it may still not include the causative gene: in our own co-expression network study (Nussbaumer et al. 2015), an F-box protein showing strong constitutive expression for lines including *Fhb1* originates from the QTL-homoeologous region on chromosome 3D. Possibly this and other genes are not present in the susceptible genotypes and thus such transcripts map to homologs or homoeologs elsewhere in the reference gene set. To unambiguously identify genes present in the QTL interval, it is therefore essential to establish the sequence of the respective genomic region in a resistant cultivar.

Here, we report sequencing and analysis of a 1 Mb genomic contig harboring *Fhb1* from the Sumai-3 derivative CM-82036. The region has been thoroughly analyzed for gene content and transcriptional activity with dense time-course RNA-seq data derived from a *F. graminearum* and mock-challenged NIL pair differing in *Fhb1*. Additional fine mapping identified recombinant lines with cross-over events mapping to the sequenced contig, thus, successfully localizing the *Fhb1* gene on a region of about 860 kb harboring 28 genes. These findings provide a relevant resource for work focused on identifying the underlying gene.

## Methods

### Generation of plant material

NILs for *Fhb1* and *Qhfs.ifa*-*5A* have been developed from a cross of the highly resistant donor cv. CM-82036 and the highly susceptible cultivar Remus (Sappo/Mex//Famos) using CM-82036 as the recurrent parent. CM-82036 originates from the cross Sumai-3/Thornbird-S and was developed in a shuttle breeding program between CIMMYT Mexico and South America. Remus is a German spring wheat cultivar with well-adapted agronomic characters, developed at the Bavarian State Institute for Agronomy in Freising, Germany (Buerstmayr et al. 2002). In the BC5F2 generation sister lines either homozygous for both resistant alleles at *Fhb1* and *Qhfs.ifa*-*5A* (CM-NIL38), susceptible alleles for *Fhb1* and resistant for *Qhfs.ifa*-*5A* (CM-NIL47) or the susceptible alleles at both QTL (CM-NIL51) were selected. The presence of the resistance QTL on 3BS was verified with marker UMN10 (Liu et al. 2006). The genotype of *Qfhs.ifa*-*5A* was confirmed with flanking simple sequence repeat (SSR) markers gwm304, barc186 and barc1 (Buerstmayr et al. 2003).

### BAC library construction

A bacterial artificial chromosome (BAC) library based on CM-82036 has been constructed from 20 g of deep-frozen fresh leave tissue harvested from 12-day-old seedlings at CNRGV-INRA (Toulouse, France). The extraction of nuclear DNA and preparation of high molecular weight (HMW)-DNA-agarose plugs to limit DNA shearing followed a protocol using sucrose extraction buffer (SEB) as described in Peterson et al. (2000) with slight modifications (given in the Supplementary File methods).

Following a partial *Hin*dIII test restriction digestion to establish the conditions yielding the highest fraction of 100–250 kb sized HMW-DNA fragments, a total of six plugs were digested with 0.4–0.7 units/mL *Hin*dIII yielding pools A, B and C. The protocols for digestions, fragment sizing and elution followed Peterson et al. (2000) with modifications (Supplementary File methods). Eluted HMW-DNA from all three pools was ligated into a *Hin*dIII linearized and dephosphorylated pIndigoBAC5 vector using T4 ligase for 10 h at 16 °C. For transformation by electroporation, 100 μL of *Escherichia coli* strain DH10B suspension was transformed with 15 μL desalted ligation product and left to recover in 2 mL SOC medium at 37 °C for 1 h. 50 μL of the suspension was plated as a control to test transformation efficiency on LB medium supplemented with X-Gal, IPTG and chloramphenicol (X/I/C). The remaining 1.95 mL was supplemented with 200 μL glycerol and aliquoted to contain about 600–1500 individual clones per pool in deep well plates. Pools were incubated for 16 h at 37 °C. From these stocks 300 μL working stocks were transferred into separate deep well plates. All stocks and aliquots were stored at −80 °C. At least 20 individual clones per pool were picked from the test plate, plasmid DNA was isolated, *Not*I digested and separated by pulsed-field electrophoresis to estimate yielding estimated average fragment sizes of 99.8, 138.1 and 163.7 kb for pools A, B and C, respectively. After this preliminary size estimation, remaining ligations from fractions B and C were transformed, characterized and pooled. Global isothermal amplification of BAC DNA was performed by amplification of 1 μL of denatured working stock with Phi29 (GenomiPhi V2 DNA Amplification Kit, GE Healthcare, Buckinghamshire, UK) at 30 °C for 2 h followed by heat inactivation of the enzyme. Phi29-amplified DNA was stored at −80 °C and diluted 1:200 for PCR screening of BAC DNA. This resource and dedicated screening tools are available upon request at http://cnrgv.toulouse.inra.fr.

### Screening, isolation, sequencing of BACs and assembly of the *Fhb1* sequence

Phi29 amplified pools were screened with published and newly generated PCR-markers (Supplementary Table S1) that were tested on genomic DNA of CM-82036 and nullisomic–tetrasomic substitution lines for chromosome 3B of Chinese Spring to ensure chromosome specificity. Novel markers were designed based on genic sequences and TE junctions from initially sequenced and annotated BACs. BAC-clone pools positive for either marker were identified based on distinct melting curve differences and/or PCR product formation on a BIO-RAD CFX386 qPCR. To isolate single BAC clones dilutions of 50 μL of the original working stock bacterial pools were plated on X/I/C qTrays and single colonies were picked and distributed into 386-well plates containing selective medium. Single colonies from overnight-incubated 386 well plates were pooled into one sample and screened for the respective marker. If these were positive the single colonies were pooled by rows and columns which were further screened to identify single candidate BACs. Isolated and validated BACs were fingerprinted by *Not*I digestion to estimate clone sizes and sequencing of BAC ends was done by Sanger sequencing. Midi-prepped (Nucleobond Xtra midi kit, Macherey–Nagel, Düren, Germany) single BAC clones were subjected to 454 sequencing (clones 235H14, 217J06, 131J06, 90G13, 114J13, 2O7, 28L19, 18G11) or 454 paired-end sequencing (305I1) at CNRGV or sequenced as one indexed sample (238O16) on a PacBio SMRT cell along with other unrelated samples by a commercial sequencing provider (GATC, Konstanz, Germany). The BAC names given here are abbreviations from the complete clone names (e.g., Tae-B-82036-ng-235H14). Raw 454 read data were processed and assembled using Newbler (version 2.7). Resulting contigs were preliminary ordered by mapping BAC end sequences and genetic markers on the contigs to the published homologous region on 3B of the reference cultivar Chinese Spring (GenBank accession: FN564434). The contig order was refined and remaining gaps were closed by sequencing equimolar pooled BACs 217J06, 235H15, 18G11, 114J13 and 90G13 on one PacBio SMRT cell (GATC, Konstanz, Germany) cells using AHA scaffolding (Bashir et al. 2012) for orientating and ordering contigs and PBJelly (English et al. 2012) for filling gaps. *Ab initio* gene models refined by BLASTx and protein domain prediction algorithms were retrieved using the semi-automated annotations pipeline TriAnnot (Leroy et al. 2012). The finished annotated contig sequence is deposited at NCBI (GenBank accession: KU641029).

### Establishing a fine map for the *Fhb1* region

In the BC5F2 generation of the NIL development (see generation of plant material), four plants heterozygous for markers spanning the *Fhb1* region (gwm493, UMN10, barc133) and homozygous for the resistant alleles at *Qhfs.ifa*-*5A* were selected, further multiplied and plants heterozygous at the *Fhb1* region were selected and harvested. 3000 of these BC5F2 lines, which are in the F2 generation for the *Fhb1* region, comprise the fine mapping population. These BC5F2 lines were grown in the greenhouse and genotyped using flanking markers gwm493 and barc133 in 2013 and 2014. Lines with recombinations between both markers, but still heterozygous for one of them, were brought to the next generation to select homozygous recombinant plants.

The recombinant NILs were further genotyped with a set of seven newly developed SNP markers using KASP-marker assays (LGC-Genomics, Middlesex, UK), based on genic sequences from the established genomic sequence covering *Fhb1* and snp3BS-8 (Bernardo et al. 2011). The original UMN10 marker from Liu et al. (2008) was replaced with a novel KASP assay derived from the sequenced UMN10 PCR product of cv. Remus. In addition, dominant gene-specific markers were developed for five of the annotated genes in the *Fhb1* region, which were scored on agarose gels. All marker data and primer sequences are collected in Supplementary Table S1.

### Greenhouse trials for FHB and DON resistance

The greenhouse experiments were conducted in 2015 as described in Steiner et al. (2009). *F. graminearum* conidia spores from strain IFA65 required for inoculation were produced on defined SNA medium under UV-light at 25 °C. After two weeks, conidia were harvested and diluted to 50,000 conidia/mL. Aliquots were stored at −80 °C. For every individual recombinant line in the fine-mapping population and the control lines CM-82036, Remus, CM-NIL38, CM-NIL47 and CM-NIL51 two pots were sown with five plants each. The experimental design was a randomized complete block design, with two replications. Temperature in the greenhouse was on average 18/12 °C (day/night) from tillering to heading with 12–14 h daylight. During flowering time, the conditions in the greenhouse were controlled and set at 22 and 17 °C during night with a 16 h photoperiod at 15,000 lux.

FHB resistance evaluations: At anthesis two central adjacent spikelets (four florets) of at least five heads per genotype and replication were inoculated by pipetting 10 µL of conidia spore suspension (500 conidia) between palea and lemma of the two basal florets. Inoculated heads were sprayed with water to provide high humidity and covered with plastic bags for 48 h. The number of diseased spikelets was counted 26 days after inoculation (dai). For each pot, the mean FHB severity as number of diseased spikelets/head was used for further analyses.

Similarly, an additional experiment was carried out to sample tissue for RNA profiling using the exact same protocol but the two NILs CM-NIL38 and CM-NIL51 only. The experimental design was a randomized complete block design with three blocks (=replications) each representing the four possible combinations of two genotypes (CM-NIL38, CM-NIL51) by two treatments (*F. graminearum*, mock) combinations. Six central spikelets were inoculated to increase the amount of uniformly treated tissue with either mock or *F. graminearum* spore suspension. Five heads were sampled for each of these conditions at 3, 6, 12, 24, 36 and 48 h after inoculation (hai) in three biological replicates (a total of 360 plants).

DON resistance evaluation: a subset of 35 recombinant NILs representing eight of the nine detected haplotypes was tested for DON resistance. The production and application of the toxin in the heads were conducted as described by Lemmens et al. (2005) with slight modifications: At anthesis, four distal florets of two central spikelets were treated once with 20 μL of a DON solution (12 g/L DON, 0.1 % Tween). Treated heads were sprayed with water and covered with plastic bags for 24 h. The number of DON-bleached spikelets was assessed 26 dai. For each pot, mean DON severity as number of DON-bleached spikelets/head was used for further analyses.

### RNA sequencing

For RNA profiling, frozen tissue samples were ground under sterile conditions and pooled to comprise a single sample/data point as described in Kugler et al. (2013). 100 mg frozen tissue was used to extract RNA using the RNeasy Plant Mini Kit (Qiagen, Venlo, Netherlands). Quality and quantity were checked on an automated electrophoresis-system (Experion, #701-7000, Bio-Rad, Hercules, CA, USA). Samples were sequenced on Illumina HiSeq 2000/MiSeq machines (Eurofins MGW, Ebersberg, Germany) yielding at least 20 M 100 bp paired-end reads per sample (Supplementary Table S2). The respective data sets are available in the EBI ArrayExpress (http://www.ebi.ac.uk/arrayexpress/) repository under the accession number E-MTAB-4222. Tophat (Trapnell et al. 2012) was used to assign reads from the susceptible and resistant genotype to the *Fhb1* contig (Supplementary Table S2). Reads were kept which showed at maximum one error in the alignment over its entire read length. Next, the contig-mapped reads were compared to the same data set mapped to the high confidence and low confidence gene sets generated from all 21 chromosomes of Chinese Spring (Mayer et al. 2014) to detect falsely mapped reads. Reads were removed from the contig alignment when they found better matches to contigs from 20 Chinese Spring chromosomes excluding chromosome 3B. HTSeq (Anders et al. 2015) was used to extract the reads counts for annotated gene models. Significant differences between treatments and lines were obtained using the exactTest function in the R package EdgeR (Robinson et al. 2010).

SNP in CDS regions were detected by searching for polymorphisms present only in mapped reads from CM-NIL51, which includes the Remus susceptible *Fhb1* region compared to reads originating from the CM-82036 *Fhb1*-region (CM-NIL38). We used the software package Geneious 8.1.7 (Kearse et al. 2012) to call SNP covered by at least five reads.

## Results

The resistant locus of *Fhb1* includes a 395 kb highly dissimilar sequence compared to the region in the susceptible Chinese Spring reference.

**Fig. 1:**
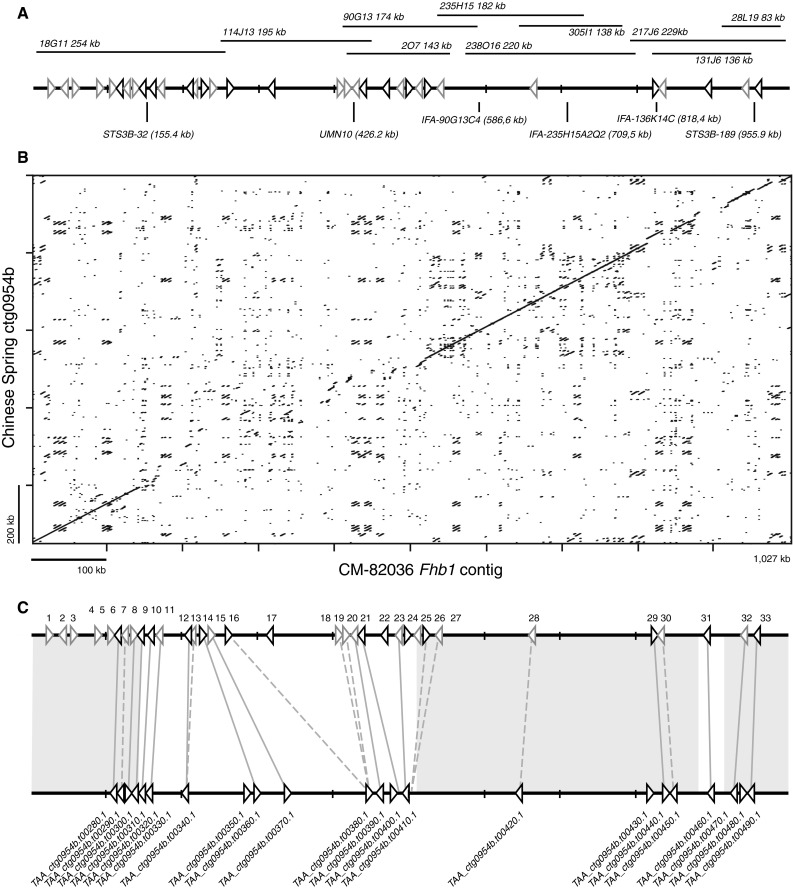
Reconstruction of the *Fhb1* region in CM-82036. **a** Sequenced BACs bridge a distance of 1.027 kb including flanking markers sts32 and sts189 and near-diagnostic marker UMN10. *Arrows* indicate positions and orientations of annotated genes. *Light gray arrows* relate to low confidence gene models. **b** *Dot plot* analysis comparing the sequenced *Fhb1* contig to the colinear segment of the published sequence of Chinese Spring (ctg0954b). **c** Comparison of gene content between CM-82036 and Chinese Spring. Regions with highly similar genes are shaded in *gray*. Genes with homology <80 % are indicated by *dashed lines*

We have established a BAC library based on the *Fhb1* donor line CM-82036 yielding 488,390 clones (576 pools) with a mean insert size of clones of 146 kb, corresponding to a total coverage of 3.9 genome equivalents. The library was first screened for BACs harboring flanking markers sts32 and sts189 (Liu et al. 2006, 2008) or the near-diagnostic UMN10 (Liu et al. 2008), which yielded several BACs (18G17, 114J13, 2O7, 90G13) that bridge the distance between sts32 and UMN10, but not between the latter and sts189 for which one BAC was isolated (28L19, Fig. 1a). Additional subgenome-specific markers (Supplementary Table S1) based on transposable element-junctions and genic sequences were generated from BAC end sequences or fully sequenced BACs. Ultimately, ten BACs of which five cover the entire contig were sequenced and assembled to form a contig of 1029 kb (Fig. 1a, GenBank accession: KU641029).

The distance between flanking markers sts32 and sts189 stretches 800.5 kb compared to 752 kb of the same interval in the Chinese Spring. While markers sts32 and UMN10 are evenly spaced in both cultivars, the distance between UMN10 and sts189 is around 49 kb larger in CM-82036. A dot plot analysis using a 100 bp word size to compare collinearity between the *Fhb1*-contig and the respective region in Chinese Spring showed more extensive rearrangements. Both sequences are highly dissimilar between positions 143 and 517 kb of the *Fhb1*-contig, which also includes UMN10 at position 426.0 kb, but high similarities exist in the flanking regions (Fig. 1b). Two gaps (positions 860.7 and 888.3 kb) remain unresolved after 454 sequencing of either BAC and additional PacBio sequencing. Both gaps are located in annotated transposable elements and are covered by the same two BACs, 217J6 and 131J6. The missing sequences are most likely not longer than several kb each and remain part of the flanking transposons (Supplementary File Fig. S1). The sequence lengths of the finished BAC sequences of BACs 217J6 (229 kb) and 131J6 (136 kb) are matched well by size estimates of restriction endonuclease-digested BAC clones (235 and 135 kb, respectively, Supplementary File Fig. S2). While the gap at position 860.7 kb is part of a segment not present in Chinese Spring, the distance between flanking sequence of the gap at 888.3 kb spans 4.3 kb in Chinese Spring.

### Unique genes in the *Fhb1* locus

Thirty-tree genes were identified in the contig by TriAnnot (Leroy et al. 2012) (Table 1; Fig. 1c). Of these, 14 gene models were classified as high confidence, with clear biological evidences for start/stop codons and intron–exon junctions. Two low confidence genes (#13 and #19) located in the 5′-UTR or N-terminal regions of genes #12 and #18, respectively, most likely represent erroneous gene calls. A BLASTn search for the best matches against the genomic contig ctg0954b covering the *Fhb1* region in Chinese Spring and against the IWGSC 2.2 high and low confidence gene sets identified the best matches against the Chinese Spring reference (Table 1; Fig. 1c; Supplementary Table S3). Here, we found that in accordance with the direct comparison of genomic sequences also the gene content in the *Fhb1* region is different to Chinese Spring. With the exception of four low confidence gene models located on the very distal end of the sequenced region (genes #1–#4 in contig range 25–128 kb), genes in the flanking sections of the sequenced genomic region match genes annotated on ctg0954b well: genes #6–#9 correspond to TAA_ctg0954b.00280.1–TAA_ctg0954b.00310.1 and genes #28–#33 match TAA_ctg0954b.00420.1–TAA_ctg0954b.00490.1. For two additional low confidence genes in Chinese Spring TAA_ctg0954b.00430.1 and TAA_ctg0954b.00480.1, encoding an unknown protein and an F-box domain-containing protein, no evidence was found that either gene is also present in the *Fhb1* region.

**Table 1:** Genes located in the *Fhb1* region of CM-82036

Relative position	Start position	End position	Gene model confidence	Final annotations	Genbank protein ID	Best BLASTN hit against ctg0954b^a^	Start position	End position	*e* value	Coverage (%)
1	24,668	27,579	Low	B3 DNA binding domain	AML47749	No gene annotated	2,072,979	2,073,310	6.59e−172	36.64
2	48,535	45,853	Low	Uncharacterized protein	AML47750	No gene annotated	2,092,786	2,092,483	3.35e−154	53.33
3	56,213	60,975	Low	Uncharacterized protein	AML47751	No gene annotated	2,102,842	2,103,174	1.66e−165	47.44
4	78,026	92,795	Low	Coatomer subunit beta	AML47752	No gene annotated	2,133,657	2,134,075	0	12.55
5	107,041	122,851	Low	Protein kinase domain (3.2e−66)	AML47753	No gene annotated	2,162,602	2,163,434	0	19.24
6	127,969	126,494	High	Glycosyltransferase HGA-like	AML47754	TAA_ctg0954b.00280.1	2,174,053	2,172,578	0	100.00
7	136,773	128,890	Low	Leucyl-tRNA synthase	AML47755	TAA_ctg0954b.00290.1	2,182,639	2,174,973	0	81.45
8	139,997	140,842	Low	Exonuclease	AML47756	TAA_ctg0954b.00300.1	2,185,258	2,186,109	0	100.00
9	144,608	141,782	High	Alanyl-tRNA synthase	AML47757	TAA_ctg0954b.00310.1	2,189,865	2,187,012	0	100.00
10	155,626	154,585	High	Uncharacterized protein	AML47758	TAA_ctg0954b.00320.1	2,194,051	2,193,014	0	100.00
11	163,946	162,916	Low	PAP fibrilling domain-containing protein	AML47759	TAA_ctg0954b.00330.1	2,205,687	2,204,656	0	100.00
12	206,176	201,561	High	tRNA (guanine-*N*(1)-)-methyltransferase	AML47760	TAA_ctg0954b.00340.1	2,252,251	2,248,125	0	98.55
13	206,638	206,375	Low	tRNA (guanine-*N*(1)-)-methyltransferase	AML47761	TAA_ctg0954b.00340.1	2,252,251	2,248,125	2.38e−57	50.00
14	210,073	212,023	High	Polygalacturonase	AML47762	TAA_ctg0954b.00360.1	2,298,469	2,300,408	0	100.00
15	225,343	228,742	Low	Oxidoreductase NAD-binding domain	AML47763	TAA_ctg0954b.00370.1	2,338,991	2,341,411	0	99.34
16	275,842	279,821	High	Terpene synthase	AML47764	TAA_ctg0954b.00380.1	2,447,582	2,450,661	0	59.73
17	317,739	317,218	High	Ubiquitin-2 like Rad60 SUMO-like	AML47765	No hit				
18	404,453	404,671	Low	Delta-cadiene synthase	AML47766	TAA_ctg0954b.00380.1	2,447,582	2,450,661	1.95e−38	45.66
19	404,803	407,686	Low	Terpene synthase	AML47767	TAA_ctg0954b.00380.1	2,447,582	2,450,661	0	64.21
20	409,497	408,408	Low	Hypothetical protein	AML47768	TAA_ctg0954b.00390.1	2,461,316	2,460,534	0	90.48
21	431,117	430,236	High	General transcription factor IIE subunit	AML47769	No gene annotated	2,477,597	2,476,727	0	98.75
22	463,855	460,654	High	Agglutinin	AML47770	No hit				
23	490,689	488,929	Low	E3 ubiquitin-protein ligase	AML47771	TAA_ctg0954b.00410.1	2,496,701	2,494,320	0	83.16
24	492,628	493,975	High	GDSL lipase acylhydrolase	AML47772	No hit				2.71
25	510,096	509,740	Low	Cystatin	AML47773	No hit				
26	518,817	520,031	High	F-box domain-containing protein	AML47774	No gene annotated	2,504,286	2,504,808	0	43.05
27	530,112	529,579	Low	Hypothetical protein	AML47775	No gene annotated	2,518,849	2,518,633	3.49e−101	56.96
28	655,286	648,498	Low	Eukariotic translation initiation factor	AML47776	TAA_ctg0954b.00420.1	2,649,048	2,642,328	0	83.23
29	833,839	837,490	High	Methyltransferase domain-containing protein	AML47777	TAA_ctg0954b.00440.1	2,839,635	2,843,304	0	100.00
30	840,463	838,212	Low	Zinc finger C3H4 type (RING finger) domain-containing protein	AML47778	TAA_ctg0954b.00450.1	2,846,282	2,843,886	0	82.22
31	899,202	897,561	High	Cytochrome P450	AML47779	TAA_ctg0954b.00460.1	2,902,331	2,900,715	0	100.00
32	946,118	943,395	Low	NB-ARC domain-containing protein	AML47780	TAA_ctg0954b.00470.1	2,935,328	2,931,924	0	99.67
33	960,993	955,862	High	Uncharacterized protein	AML47781	TAA_ctg0954b.00490.1	2,950,583	2,945,429	0	100.00

Relative positions in the direction of markers sts32–sts189

^a^BLASTn against CDS sequences of ctg954b. If no hits were retrieved sequences were additionally used as query to search the ctg954b genomic DNA sequence, yielding “no gene annotated” as a positive or “no hit” as negative result

The central section does—despite the high sequence divergence to Chinese Spring—harbor several genes present in both genotypes. Most of these are located in a segment between sts32 and the central marker UMN10: six genes (#10–#15, between positions 155 and 228 kb) are annotated in both cultivars. Additionally, Chinese Spring harbors TAA_ctg0954b.00350.1, encoding a C-terminally truncated UDP-glucose dehydrogenase (lacking active site residues), which is not present in the *Fhb1* region of CM-82036.

A second segment between marker UMN10 and sts189 hosts 12 genes (#16–#27, positions 288–530 kb) of which only few find best matches to the annotated genes on ctg0954b. These share overall poor coverage and similarity to the BLASTn matched genes. None of the six predicted high confidence genes in this segment finds matches to 3B-mapped IWGSC high confidence genes and only one finds a match in the low confidence gene set (Supplementary Table S3). Several, however, find best matches to putative homoeoallelic loci on chromosomes 3D (#26 and #27) and 3A (#17), while others (#22, #24 and #25) share high similarities to genes located on chromosomes 2A, 2B and 4D, respectively. In contrast, the Chinese Spring sequence contains one gene, TAA_ctg0954b.00400.1 (unknown), which is not predicted by TriAnnot on *Fhb1*. Yet two of the four ab initio pipelines employed by TriAnnot suggest a gene model, which finds no further evidence in database comparisons to expressed genes.

At the distal end of the sequenced contig around marker sts189 genes #28 to #33 find best matches in the Chinese Spring reference sequence. The *Fhb1* region seems to lack TAA_ctg0954b.t00480.1, an F-box domain-containing protein located between genes #32 and #33, for which no gene models were predicted. This highly colinear region to Chinese Spring also covers the two remaining small gaps in the sequenced contig with no predicted Chinese Spring genes mapping therein.

### Gene expression profiles in the *Fhb1* region in response to *F. graminearum*

We added expression data to the sequenced contig to further substantiate gene predictions and to gain an impression of expression dynamics in this region in the presence or absence of the pathogen. Expression profiles were generated from 72 RNA-seq libraries originating from *F. graminearum* and mock-inoculated wheat head tissues sampled in a dense time-course series from three to 48 h after inoculation. The sampled tissue derives from a newly developed NIL pair with the resistance donor CM-82036 as the recurring parent containing either *Fhb1* and *Qfhs.ifa*-*5A* (CM-NIL38) or susceptible alleles originating from the German spring wheat cultivar Remus (CM-NIL51). To account for polymorphisms in CM-NIL51 to the sequenced contig, we allowed one mismatch per read when mapping reads to the *Fhb1* region. We controlled falsely mapped reads by mapping reads also against the IWGSC high and low confidence gene sets. About 10 % of the mapped reads found better matches in these data sets excluding genes from chromosome 3B and were excluded from any further analysis.

**Fig. 2:**
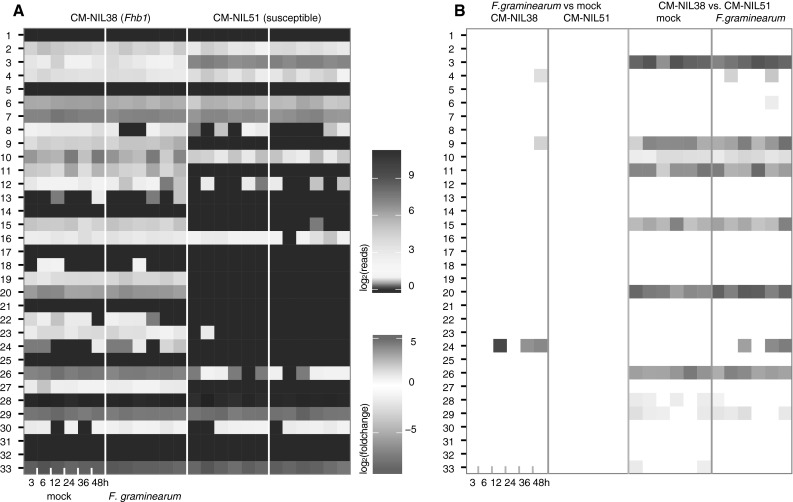
Expression analysis of genes located in the *Fhb1* region of CM-82036. **a** log_2_-transformed RNA-seq read counts for genes located in the region. Each *subpanel* comprises time-course-derived data (3–48 h) after inoculation with *F. graminearum* or mock for either the *Fhb1*-carrying CM-NIL38 or the susceptible CM-NIL51. Genes with no mapped reads are given in *dark blue*, highly expressed genes in *red*. **b** Differentially expressed genes in contrasts comparing *F. graminearum*-challenged to mock-treated samples in the resistant CM-NIL38 and the susceptible CM-NIL51 (*left panels*) and comparing mock-treated and *F. graminearum*-inoculated samples between the two NILs (*right panels*). Positive log_2_-transformed fold-change values indicate significant higher expression in response to the pathogen (*left panels*) or higher expression for the susceptible CM-NIL51 when comparing similarly treated samples between NILs (*right panels*). White spaces represent samples with no significant (FDR >0.05) differences (yet differences in read counts as indicated in **a** might occur) (color figure online)

Despite these considerations, only few genes in the central segment of the sequenced contig were hit by reads originating from the susceptible CM-NIL51 (genes #8–#27), while genes in the flanking non-divergent regions are generally expressed at comparable levels in either NIL (Fig. 2a; Supplementary Tables S4 and S5 including raw read counts and significant changed genes, respectively). Other large constitutive expression differences have only been detected for gene #3, encoding a protein of unknown function, for which we find higher transcript abundances in the susceptible CM-NIL51.

Genes #10 and #11, both coding for unknown proteins, show a circadian-type expression with expression peaks at 3, 24, and 48 hai (representing morning hours) in the resistant CM-NIL38, while in the susceptible CM-NIL51 only gene 10 shows a similar expression pattern albeit to a much weaker extent (Fig. 2a). None of the genes in the susceptible NIL are significantly different expressed in response to the pathogen and only three show significant changes in expression in the resistant NIL (Fig. 2b): transcripts corresponding to gene #4 encoding a coatamer subunit domain-containing protein (G4) were 3.61-fold less abundant at 48 hai in the *F. graminearum*-challenged samples compared to mock, while at the same time point transcript abundances for gene #9, encoding an alanyl-tRNA synthase, were 2.25-fold increased in response to the pathogen. Striking differences were observed for gene #24 encoding a GDSL lipase (acylhydrolase): significantly higher transcript abundances in *F. graminearum*-treated samples were detected at 12, 36 and 48 hai and a 15.76-fold expression increase at 48 hai was observed.

### Fine-mapping *Fhb1*

**Fig. 3:**
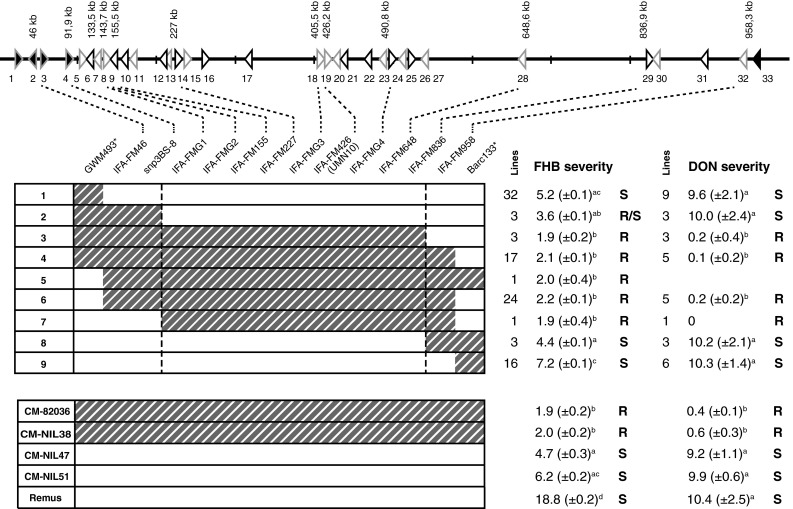
Graphical illustration of the nine haplotypes and control lines in the *Fhb1* interval for 16 markers and their phenotypes for FHB and DON resistance. Resistant (CM-82036) alleles for the markers are illustrated by *dashed boxes*, whereas susceptible (Remus) alleles are shown in *white*. The FHB/DON resistance levels are measured in number of diseased/DON-bleached spikelets per head 26 days after *F. graminearum*-inoculation or DON infiltration. The *dotted lines* define the interval harboring *Fhb1*. Resistant (*R*) and susceptible (*S*) haplotypes significantly different from each other are indicated by the *respective* *letters* (FHB severity: mean LSD *α* = 5 % = 2.0, DON severity: mean LSD *α* = 5 % = 3.0)

A large mapping population was developed to genetically narrow down the *Fhb1* region and thereby reducing the number of *Fhb1* candidates. Polymorphisms for marker design were detected utilizing mapped RNA-seq data from the susceptible Remus to the *Fhb1* region of CM-82036. Using only SNPs unique to CM-NIL38-mapped reads with a frequency of >90 %, we detected 30 polymorphisms within predicted coding sequences, which comprise a conservative estimate of SNP in the region (Supplementary Table S6). We used these and SNP identified by mapping reads from a previous RNA-seq project using similar NILs (Kugler et al. 2013) to design six KASP genotyping assays. An additional KASP assay was constructed based on resequencing the PCR product of UMN10 from the susceptible and highly polymorphic locus of cv. Remus (Supplementary File Fig. S3). To design markers for regions not covered by RNA-seq due to the absence of genes in the susceptible parent, we generated four PCR assays that produce a dominant-type PCR amplicon from annotated genes found only in the *Fhb1*-region of CM-82036. All together 14 markers separated the 100 recombinant NILs in nine haplotypes, whereas nine of the markers co-segregated resulting in a 703.4 kb region, between contig positions 133.5 and 836.9 kb, with no recombination events found within (Fig. 3; Supplementary File Fig. S4, which includes the number of inoculated heads).

Disease severity separated CM-82036 and the near-isogenic control lines into two phenotypic classes depending on the presence of *Fhb1*. The control lines, possessing *Fhb1*, developed disease symptoms on the two inoculated spikelets per spike only; no further spreading of disease symptoms was observed, whereas the NILs with susceptible alleles at *Fhb1*, CM-NIL47 and CM-NIL51, showed disease symptoms in 4.7 and 6.2 spikelets/head on average. The FHB severity of the highly susceptible cultivar Remus was three times higher as observed for the susceptible NILs.

Statistical analysis of the 100 recombinant NILs representing nine haplotypes detected three phenotypic classes: for the FHB-resistant class (haplotypes 3, 4, 5, 6, 7) just the two point-inoculated spikelets per head exhibited disease symptoms, as also observed for the resistant control lines. These lines have CM-82036 alleles for nine markers in common, from contig position 133.5–836.9 kb. The FHB susceptible haplotypes (1, 8, 9) were grouped in two phenotypic classes, with haplotype 9, being slightly more diseased with on average 7.2 symptomatic spikelets per spike compared to haplotypes 1 and 8 with 4.4 and 5.2 diseased spikelets. Haplotype 2, representing lines with recombinations between snp3BS-8 and IFA-FMG1 and CM-82036 alleles for snp3BS-8, but susceptible alleles at IFA-FMG1 exhibited an intermediate FHB resistance phenotype, being not significantly different from the resistant and the susceptible class. The mean of 3.6 symptomatic spikelets/spike rather points towards the susceptible class.

In addition, a subset of the recombinant NILs and the control lines were tested for DON resistance after application of the toxin DON in the head. The toxin treatment to the flowering heads induced typical FHB symptoms, straw-like color, spreading in both acropetal and basipetal directions, only in DON-sensitive lines (Supplementary File Fig. S5). DON severity (measured as number of DON-bleached spikelets per spike 26 days after treatment) divided all tested lines into two distinct phenotypic classes: the DON resistant class with almost no symptoms and the DON susceptible class with about ten DON-bleached spikelets per spike. The DON resistant class comprises haplotypes 3, 4, 6, and 7. The DON susceptible class with haplotypes 1, 2, 8 and 9 positioned *Fhb1* in the same contig interval as obtained for FHB severity, with the flanking markers snp3BS-8 and IFA-FM958 mapping to the contig positions 91.9 and 958.3 kb, respectively. The separation of all lines, including control lines and the highly susceptible cultivar Remus into two distinct classes, identifies *Fhb1* as the only gene for toxin resistance in CM-82036.

We could successfully connect the physical map of the *Fhb1* region to the genetic map; the two markers, snp3BS-8 and IFA-FM958, place the causal gene(s) behind *Fhb1* between the contig positions 91.9 and 958.3 kb, respectively. One or several genes in this region confer FHB and DON resistance.

## Discussion

We have determined the genomic sequence of the wheat FHB resistance QTL *Fhb1* in the resistant donor cultivar CM-82036 and positioned the gene within a region covering 860 kb using a fine-mapping panel for *Fhb1* that has been phenotyped for both FHB and DON resistance. 28 candidate genes including 13 high confidence genes located in this interval have been further characterized in a dense time-course RNA-seq study.

### Suppressed recombination in the highly divergent region of *Fhb1*

Our findings show that the sequence containing *Fhb1* in CM-82036 differs significantly from the susceptible Chinese Spring reference in gene content and size. The core region of the sequenced contig is highly dissimilar to Chinese Spring when comparing genomic DNA; also markers designed for this region in CM-82036 failed to amplify in the susceptible region of cv. Remus. A dot plot analysis did not identify structural rearrangements such as genomic inversions or duplications (Fig. 1b). The in part large differences in distance between the genes present in resistant and susceptible cultivars may be attributed to different transposon insertions events (Scherrer et al. 2005). Most genes unique for *Fhb1* reside in a compact cluster (genes #18–#27). The observed differences can be explained in part by pseudogenisation of genes #21, #26 and #27 in Chinese Spring where partial overlaps were still identifiable in non-annotated regions. However, multiple genes are unique for either the *Fhb1* contig or Chinese Spring.

Haplotype divergences and loss of microcolinearity between cultivars on a comparable scale as detected for *Fhb1* have been observed several times before (i.e., for the barley *Rph7* locus (Scherrer et al. 2005) and wheat *Lr10* (Isidore et al. 2005)) and also in two recent studies: Yeo et al. (2015) have resequenced the resistant and susceptible loci from two barley cultivars differing in the *Puccinia hordei* resistance gene *Rphq2* and identified entirely different haplotypes of which the resistant locus harbors unique candidate genes. Mago et al. (2014) established the genomic region harboring the wheat stem rust resistance gene *Sr2*, which includes a cluster of germin-like proteins missing in the susceptible reference cultivar Chinese Spring. The authors showed that this cluster is shared between other resistant accessions containing *Sr2*.

In rice, more than 6 % of the genome is occupied by regions of high divergence (Tang et al. 2006). The higher genome plasticity of polyploid wheat led to a high rate of gene deletions and activity of repetitive elements buffered by the redundancies within the three homoeoallelic subgenomes (Dubcovsky and Dvorak 2007). Most likely the rate of highly divergent regions between cultivars is more frequent than generally assumed. Redundant gene content may also more easily accommodate introgressions of rare resistance haplotypes from landraces under selective pressure by replacing genes with redundant gene activity.

Highly diverging haplotypes generally lead to strongly reduced meiotic recombinations in such regions. In the *Fhb1* locus, loss of colinearity has a direct effect on recombination frequency. While flanking recombining regions harbor 4 and 6 recombinations in an interval of 42.6 and 122 kb, respectively (Fig. 3) about 700 kb remain unresolved. On a larger scale, the locus itself resides in a highly recombining telomeric region of chromosome 3B with an average of 0.85 cM/Mb (Saintenac et al. 2009), which would relate to 17 expected recombinations within the *Fhb1* non-recombining region. Tracking recombinants from two mapping populations of which neither parent harbored *Fhb1* the same authors detected a recombination hotspot that covers the region around UMN10 in one of their populations leading to high recombination rates, while only a below average rate was observed in the second population (Saintenac et al. 2011). Apparently, cross-over hotspots exist in this region, but these need to be met with matching crossing partners.

As CM-82036 is a direct derivative from a cross of the *Fhb1* donor Sumai-3, the sequence obtained from this cultivar should be directly comparable to the sequence of Sumai-3, which has been target of several fine-mapping studies (Cuthbert et al. 2006; Liu et al. 2008; Bernardo et al. 2011). Among the markers used in both studies, the widely used UMN10 is the only marker we found mapping within the non-colinear region, while all others mapped to flanking regions. The original UMN10 from Liu et al. (2008) detects a length polymorphism. This marker has been converted into an easily applicable KASP-SNP assay, which has been successfully employed on diverse germplasm conducted by several groups so far and should remain a reliable marker for broader use.

The closest reported recombination event is based on a single recombinant line for marker sts32 reported by Liu et al. (2008) which mapped to position 155.4 kb on the *Fhb1* contig. Consideration of this single event would exclude genes #1–#10 from the list of *Fhb1* candidates. To further substantiate these findings, we identified 100 recombinant lines in our own fine-mapping panel for the gwm493 and barc133 interval. Yet, we failed to identify additional recombinant lines that would reach as far or further in the ‘core’ region of *Fhb1*.

### Candidate genes in the *Fhb1* locus

*Fhb1* expresses a dominant phenotype (Xie et al. 2007 and own unpublished data). Consequently, possible explanations for the phenotypic difference could be induced expression of the underlying gene in response to the pathogen or constitutive expression in the resistant genotype, or absence of the respective gene in susceptible lines as Chinese Spring and Remus. Furthermore, gain of function polymorphisms through changes in protein sequence may cause the resistance phenotype. Also susceptibility factors encoded in the interval in lines lacking *Fhb1* need to be considered, although such a scenario is more difficult to reconcile with the reported dominance. Our results also show that DON resistance (determined as bleaching resistance after application of high concentrations of pure toxin) is clearly associated with *Fhb1*. Either DON resistance itself could simultaneously lead to FHB resistance, or the gene causing DON resistance might be tightly linked to the gene conferring FHB resistance by a different mechanism. The fact that increased *F. graminearum* resistance was achieved by increasing DON resistance [due to overexpression of a barley glucosyltransferase (Li et al. 2015)] suggests that genes with an effect on toxin resistance should be considered as prime candidate *Fhb1* genes. With the sequenced region at hand and mapped expression data, these scenarios can now be considered much better; albeit lacking a higher resolved map still many candidate genes remain: We discuss putative functions and expression patterns of the candidate genes and the implications for functional testing.

Gene #17 (ubiquitin-2 like Rad60 SUMO-like protein) is unique for the CM-82036 sequence, deleted in the susceptible cultivar (see Fig. 1c). In yeast, it has been shown that reducing the ubiquitin pool by disruption of the stress responsive polyubiquitin gene leads to reduced DON resistance of *ubi4* mutants (Abolmaali et al. 2008). Yet, gene #17 is practically not expressed, neither in the control nor following *F. graminearum*-inoculation. Similarly, genes #21 (general transcription factor IIE subunit) and #25 (cystatin) are not present in the susceptible Chinese Spring reference, but also not expressed under both conditions. We, therefore, consider them unlikely candidates for *Fhb1*.

All other genes within the diverging region are expressed in the *Fhb1*-containing CM-NIL38 but not in the susceptible CM-NIL51. Expression levels range from few reads per sequenced sample to hundreds of reads per sample. While highly expressed genes may present themselves as ‘more-likely’ candidates, comparably lowly expressed genes should not be ruled out as RNA levels may not directly reflect protein expression levels and the abundances to establish a specific function may be vastly different for individual gene products.

Gene #19 (terpene synthase), gene #20 (unknown protein) and gene #23 (E3-Ubiquitin ligase) are also present in Chinese Spring but they are only expressed in *Fhb1* containing lines. Terpene synthases act in the biosynthesis of secondary metabolites, which play a role in defense against herbivores or pathogens (Lange 2015). Many terpenoid phytoalexins from *Poaceae* have been described (Ejike et al. 2013). These include phytoalexins derived from monoterpenes and sesquiterpenes, which have a direct antimicrobial effect (Schmelz et al. 2011; Inoue et al. 2013). Sesquiterpenoid phytoalexins active against *F. graminearum* have for instance been described in maize (Huffaker et al. 2011). Volatile terpenes also may act as messengers upon pathogen attack (Nagegowda 2010). The *Fhb1*-associated terpene synthase most likely acts in synthesis of cytosolic sesquiterpenoids from farnesyl diphosphate as a BLASTp result suggests (delta-cadinene synthase isozyme A, *e* value = 0). However, the overall low expression level of the terpene synthase encoded on *Fhb1*, with no observable differences between *F. graminearum* treatment and mock, suggests that this gene does not play an active role in the response to the pathogen.

A secondary annotation for gene #20 (unknown protein) suggests a role in calcium sensing (sarcoplasmic reticulum histidine-rich calcium-binding protein precursor, blastp, *e* value = 8e−38) and, consequently, may lead to changes in gene expression following external cues such as abiotic and biotic stresses (Reddy et al. 2011). Yet, its strong constitutive expression suggests a different role for this gene. The lack of expression in the susceptible NIL is not due to pseudogenisation of the CS ortholog. Both gene models seem intact and share high amino acid sequence similarity (95.7 %, Supplementary File Fig. S6). In contrast, the promotor regions are highly divergent in the first 1 kb upstream of the short 5’UTR region with multiple sequence deletions in the *Fhb1*-contig, which most likely are responsible for the observed large expression differences. Despite the lack of clear indications about its potential mode of action, gene #20 should be considered a candidate for *Fhb1*.

Gene #23 is a predicted E3-ubiquitin ligase of the ‘seven in absentia’ (SINA) type. Such proteins mediate ubiquitination and proteasome-mediated degradation of specific proteins (in response to a stimulus). Some SINA proteins and their client proteins have been implicated in plant–pathogen and plant–symbiont interactions (Kim et al. 2006; Den Herder et al. 2012). They contain an N-terminal RING domain and a C-terminal conserved domain implicated in dimerization and substrate binding. Interestingly, the gene models for this protein differ largely due to an internal deletion of 31 nt in the *Fhb1* reading frame compared to the gene model in Chinese Spring. The consequence is a frameshift and premature stop codon, removing the entire SINA domain. The *Fhb1*-resistant line, therefore, possesses most likely a nonfunctional version of the protein. Yet, the expression is higher in the *Fhb1* background, so potentially the truncated protein might act in a dominant negative fashion, so that it may be premature to exclude this gene as a candidate.

### The *Fhb1* region hosts four clearly expressed genes absent in the susceptible reference

Gene #22 encodes protein with domains encoding agglutinin and ‘pore-forming toxin-like’. This weakly expressed gene might have direct antifungal activity by binding to fungal cell wall carbohydrate structures and permeating membranes. The role of lectins in plant defense is well established (Lannoo and Van Damme 2014). Wheat germ agglutinin has been shown to bind to *N*-acetyl-d-glucosamine (Levy 1979), a monomer of the fungal cell wall chitin and as such constitutes a pathogen recognition mechanism that elicits further, early defense responses. Wheat germ agglutinin exhibits also a negative effect on hyphal growth of various fungi including *F. graminearum* (Mirelman et al. 1975; Ciopraga et al. 1999). This proposed mechanism is, however, more consistent with type I than type II resistance against spreading of the disease and resistance to DON. Expression of the gene #22 cDNA in *Saccharomyces cerevisiae* under control of the inducible *GAL1* promoter did not affect the growth of the transformed yeast strain on galactose medium (data not shown).

Gene #24 encoding a GDSL lipase is the only gene in the sequenced contig that exhibits a significant increase in expression in response to the pathogen. GDSL lipase/esterases comprise a structurally diverse gene family in plants. For instance, 114 members exist in the rice genome (Chepyshko et al. 2012). They act in regulation of a variety of physiological functions including defense. A chain of studies (Kwon et al. 2009; Kim et al. 2014) demonstrated the role of an *Arabidopsis thaliana* GDSL lipase 1 in modulating systemic immunity through the regulation of ethylene signaling in response to necrotrophic pathogens. However, the present GDSL lipase does not share similarity with the *A. thaliana* lipase 1 gene. The expression pattern and its possible role in defense warrant further investigations.

Gene #26 (F-box protein) is among the strongest constitutively expressed genes on the contig. No similar gene is annotated in this region of Chinese Spring, yet mapping of the coding sequence of this gene onto Chinese Spring identified a likely pseudogene with weak similarity (Table 1). F-box proteins are part of the ubiquitination complex, which form specific interaction with target proteins. Consequently, the gene family is very large with 779 genes in rice (Xu et al. 2009). The F-box protein could be involved in reducing the levels of protein encoding a susceptible factor for FHB. Its target protein, which is most likely not encoded in the *Fhb1* region, would need to be genetically fixed and must not segregate, to be in agreement with the absence of epistasis at *Fhb1.* Potentially, the F-box protein could also directly target an unknown effector protein of the pathogen. In *A. thaliana,* the F-box protein encoded by *COI1* is involved in jasmonate signaling and is the target of the jasmonic acid mimicking bacterial toxin coronatine which increases susceptibility (Geng et al. 2012).

Also gene #27 (hypothetical protein) cannot be excluded as gene candidate. However, only few reads map to the predicted CDS of this low confidence gene for which no annotation could be retrieved.

The genes on the right half of the *Fhb1* interval (Fig. 1c) have again counterparts in the susceptible line. The genes #28 and #29 are constitutively expressed and are discussed below. Gene #30 encodes a predicted zinc finger C3H4 type (RING finger) domain-containing protein showing low expression in both NILs, and no response to *F. graminearum* infection. Zinc finger-containing proteins have functions ranging from transcription, translation, mRNA trafficking, cytoskeleton organization, protein folding, chromatin remodeling and more. Only a domain of unknown function (DUF3675) is additionally recognized. But since the gene model is identical with that of Chinese Spring, this gene showing no significant expression difference between NILs and in response to *F. graminearum* can be excluded.

Also genes #31 and #32 are unlikely candidates due to lacking expression. A CYP450 gene could encode an enzyme involved in the biosynthesis of an antifungal metabolite, or a detoxification enzyme leading to chemical modification of the toxin structure. A bacterial cytochrome P450 detoxifying DON by hydroxylation of C16 has been described (Ito et al. 2013). The product of gene #32 contains an NB-ARC domain, which is found in plant disease resistance genes (van der Biezen and Jones 1998). Besides the nucleotide binding domain, also leucine-rich repeats can be recognized. A highly similar protein from *Aegilops tauschii* has been annotated as ‘putative disease resistance RPP13-like protein 1’ (GenBank accession: EMT27135.1). The version of the susceptible Chinese spring gene is identical in 898 of 905 amino-acids, leaving room for functional differences (Supplementary File Fig. S7). Yet, lack of expression is hard to reconcile with the otherwise suggestive role of this candidate disease resistance gene.

### How can genes on the *Fhb1* contig help explain the higher ability to inactivate DON?

Lemmens et al. (2005) have associated the *Fhb1* locus with the higher ability to metabolize DON into the non-toxic DON-3-*O*-glycoside, which is a product of the activity of toxin-specific UDP-glucosyltransferases (UGT, Poppenberger et al. 2003). No such gene is encoded on the *Fhb1* contig, gene #6 annotated as a HGA-like UGT does share similarities to the large super family encoding small molecule conjugating UGTs (Ross et al. 2001), but most likely acts on the formation of homogalacturonan (HGA) as part of the cell wall (Yin et al. 2010). Toxicity of DON is caused by inhibition of protein biosynthesis; therefore, genes involved in translation may counteract the adverse effect of DON by increasing overall translation fidelity or exerting a greater tolerance to DON in other ways. Genes #7 and #9 encode leucyl- and alanyl-tRNA synthases, respectively. While gene #7 shows no significant expression difference, gene #9 is clearly higher expressed in the *Fhb1*-containing NIL (Fig. 2) and is, therefore, more attractive. In addition, potentially relevant sequence differences exist (Supplementary File Fig. S8). Recently, it has been shown that overexpression of a methionyl-tRNA synthase from wheat when overexpressed in *A. thaliana* causes increased DON resistance in transformants (Zuo et al. 2016). Yet, as stated above, if the reported single recombinant line at sts32 (Liu et al. 2008) is indeed correct, all genes up to #10 can be ruled out as candidates. Gene #13 (tRNA-modifying methyltransferase) has a ribosome-associated function, likewise #28 (translation initiation factor). A possible role of this gene for methylation-associated resistance of ribosomes against trichothecene toxins has been proposed by Iglesias and Ballesta (1994), who found that in *Fusarium oxysporum* adaptive toxin resistance of ribosomes can be obtained by enzymatic modification of an unknown ribosomal component upon incubation with *S*-adenosylmethionin. Despite its low expression in the *Fhb1*-containing NIL, this gene should not be ruled out as candidate. Also #29 has a predicted methyltransferase domain.

With the sequence of *Fhb1* at hand the genes described in this study are a valuable resource for further functional analysis of the QTL. Based on expression profiles and annotations, some genes can be ruled out, but many remain for which further functional assessments are required. The knowledge of which gene is causing FHB resistance is not irrelevant, as breeders unknowingly may deploy proteins with potentially undesired health effects (lectin/pore-forming toxin) or increase the levels of antifungal compounds with unknown toxicological properties (terpenoid synthase). The most promising approach to further characterize *Fhb1* is the characterization of EMS-generated stable loss-of-function mutants (Slade and Knauf 2005) for which polyploid wheat is especially well suited due to the high possible mutation rates. RNA-interference methods such as VIGS may not yield clear phenotypes for targeted candidate genes as the silencing is only partial and transient. This residual expression levels bear the risk of providing sufficiently high mRNA levels to produce relevant amounts of protein to confer the resistance phenotype. Generating stable wheat transformants in a type 2 susceptible cultivar is a viable alternative to assess candidates for FHB resistance (Li et al. 2015) and should bring clarity about the gene underlying FHB and DON resistance. While the simplest hypothesis is that only one gene is causing both phenotypes, also the scenario of two different resistance genes cosegregating due to repressed recombination in the region cannot be excluded.

#### Author contribution statement

Generation of plant material: GS, BS. BAC library construction, screening and sequencing: SV, WS. Contig assembly and annotation: WS. Greenhouse trials: BS, FJ, MZ. Toxin and strain provision: ML. Marker design and genotyping: BS, VG, FJ, WS. RNAseq data acquisition and analysis: MZ, WS, TM. HB (BOKU), KFXM, GA and HB (INRA) conceived this study and obtained funding. Manuscript writing: WS, BS and GA. The manuscript was finally approved by all coauthors.

## Electronic supplementary material

Below is the link to the electronic supplementary material.

Supplementary material 1 (PDF 3321 kb)

Supplementary material 2 (XLSX 43 kb)

Supplementary material 3 (XLSX 12 kb)

Supplementary material 4 (XLSX 50 kb)

Supplementary material 5 (XLSX 48 kb)

Supplementary material 6 (XLSX 40 kb)

Supplementary material 7 (XLSX 53 kb)
