# Supplementary material for: Suppressed recombination and unique candidate genes in the divergent haplotype encoding Fhb1, a major Fusarium head blight resistance locus in wheat
Source: Theor Appl Genet. 2016 May 12;129:1607–23. doi: 10.1007/s00122-016-2727-x (PMC4943984; doi:10.1007/s00122-016-2727-x)

Supplementary material

W. Schweiger[1*], B. Steiner[1*], Sonia Vautrin[2], Thomas Nussbaumer[3,4], Gerald Siegwart[1,5], Mina Zamini[1], Florian Jungreithmeyer[1], Verena Gratl[1], Marc Lemmens[1], K.F.X. Mayer[3], Hélène Berges[2], Gerhard Adam[5], Hermann Buerstmayr[1]

Suppressed recombination and unique candidate genes in the divergent haplotype encoding *Fhb1*, a major Fusarium head blight resistance locus in wheat.

[*] these authors share first authorship

[1] Institute for Biotechnology in Plant Production (IFA-Tulln), BOKU - University of Natural Resources and Life Sciences, Konrad Lorenz Strasse 20, A-3430 Tulln, Austria

[2] French Plant Genomic Resource Centre, INRA–CNRGV, Chemin de Borde Rouge, CS 52627, 31326 Castanet Tolosan, France

[3] Plant Genome and Systems Biology, Helmholtz Zentrum München, Neuherberg, D-85764, Germany

[4] current address: Division of Computational System Biology, Department of Microbiology and Ecosystem Science, University of Vienna, 1090 Vienna, Austria

[5] Department of Applied Genetics and Cell Biology, BOKU - University of Natural Resources and Life Sciences, Konrad Lorenz Strasse 22, A-3430 Tulln, Austria

Corresponding author: W. Schweiger, wolfgang.schweiger@boku.ac.at, phone: 0043/2272/66280-205

Supplementary Methods

BAC library preparation. Differences to Peterson et al. (2000) in bold typeface.

- **Preparation of DNA-agarose plugs.** Lysis Buffer contained 1 g N Lausyl sarcosine 1g, 0.03 g Proteinase K, 0.13 g N diéthyldithiocarbamate in 100ml EDTA 0.5M ph9.1
- HMW-DNA plugs were washed in **1h in 0.5M EDTA pH9.1 at 50°C**, **1h in 0.05M EDTA pH8 at 4°C** and store at 4°C.
- **Partial digest of HMW-DNA plugs:** Plugs were washed 3x in ice cold TE 10/1 + buffer (10 mM Tris-HCl, pH8.0, 1 mM EDTA pH 8.0) phenylmethyl sulfonyl fluoride (PMSF) on ice for 1 hour per washing step and 3x in ice cold TE 10/1 on ice for 1 hour per wash to remove the PMSF.
  A serial dilution of HindIII is generated by mixing 20 µl of *Hind*III (10 units/µl) with 70 µl water and 10 µl 10X *Hind*III buffer to produce a 2.0 unit/µl *Hind*III solution. Thereof, 10 µl are added to 80 µl water and 10 µl 10X *Hind*III buffer to produce a 0.2 units/µl *Hind*III dilution. These stocks were used to produce 10 different concentrations of HindIII per sample ranging from 0.5 to 100 units/mL. Samples were incubated for **10 min at 37°C**, followed by EDTA-mediated inactivation of the enzyme.
- Sizing steps retained HMW-DNA fragment sizes between **100 kb and 250** kb.

Supplementary Fig. S1

Remaining unaccounted sequence in the *Fhb1* contig. Positions are indicated in blue. mRNA and CDS in green and yellow, respectively. Repeat elements are given in light blue. Annotations have been retrieved by TriAnnot (Leroy et al. 2012).

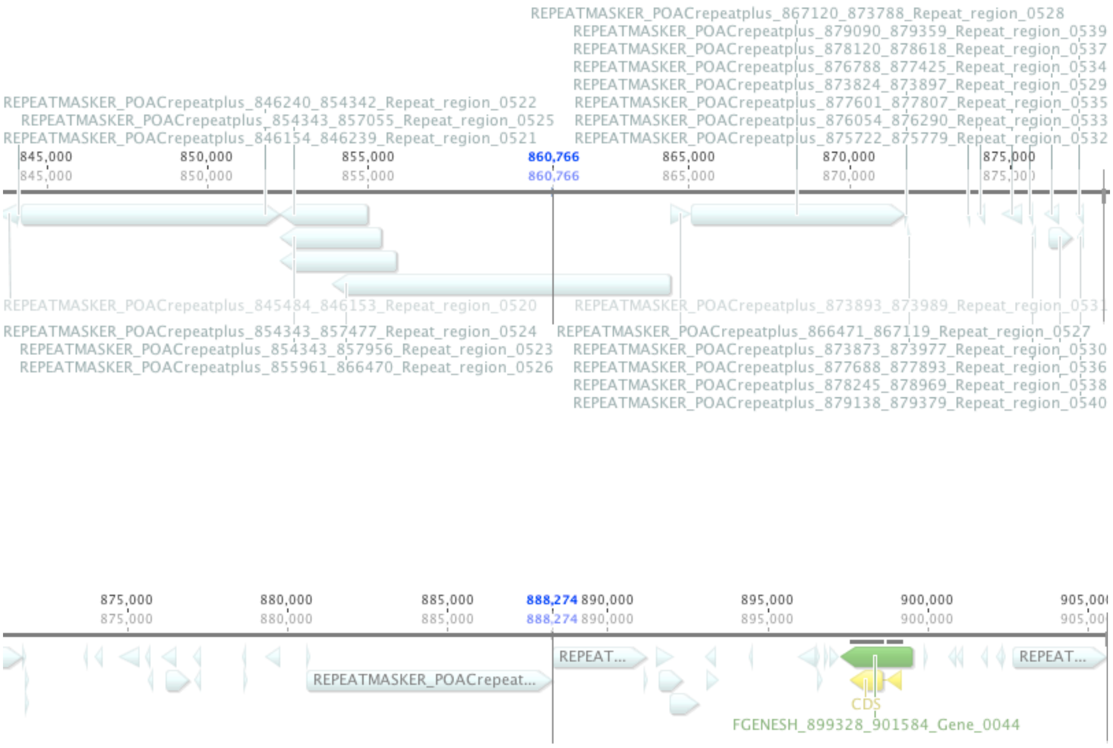

Supplementary File Fig. S2. Pulsed-field electrophoresis gels of restriction endonuclease (NotI) digest of BAC clones 217J6 (left) and 131J6 (right, 131J6-A: partial digest, 131J6-B complete digest)

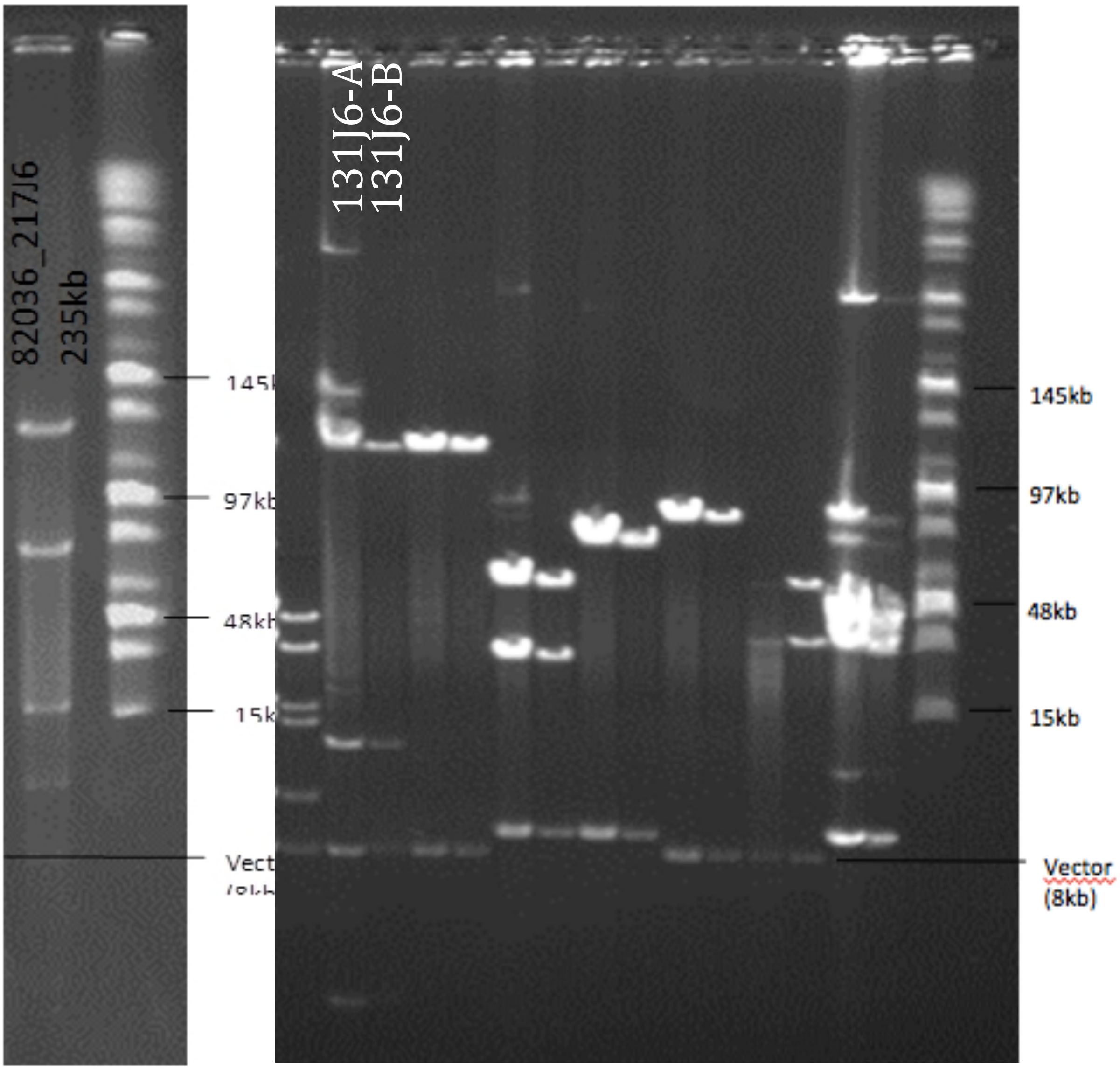

Supplementary File Fig. S3. Sequence alignment for the UMN10 locus. Resequenced PCR products from CM-82036 (*Fhb1+*, top 2 rows) and Remus (*Fhb1-* , bottom 2 rows) have been aligned to the established Fhb1 contig from CM-82036. Primers used for UMN10 (Liu et al 2008) and primers for KASP marker IFA-FM426_UMN10 are indicated.

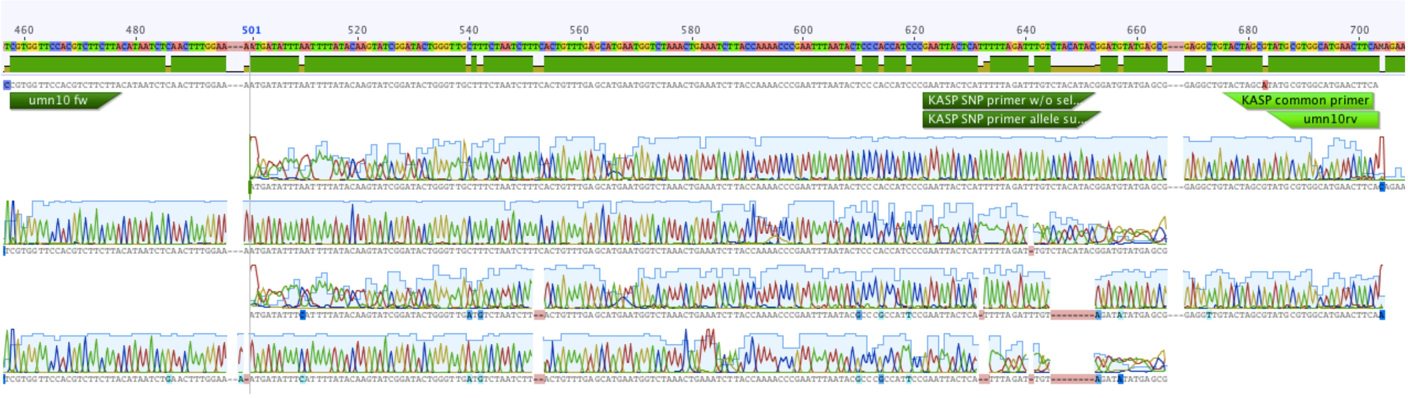

Supplementary File Fig. S4. Fine mapping of the Fhb1 region including phenotyping results for DON and FHB resistance assessment. This file is similar to Figure 3of the manuscript, but additionally includes the number of inoculated heads per haplotype.

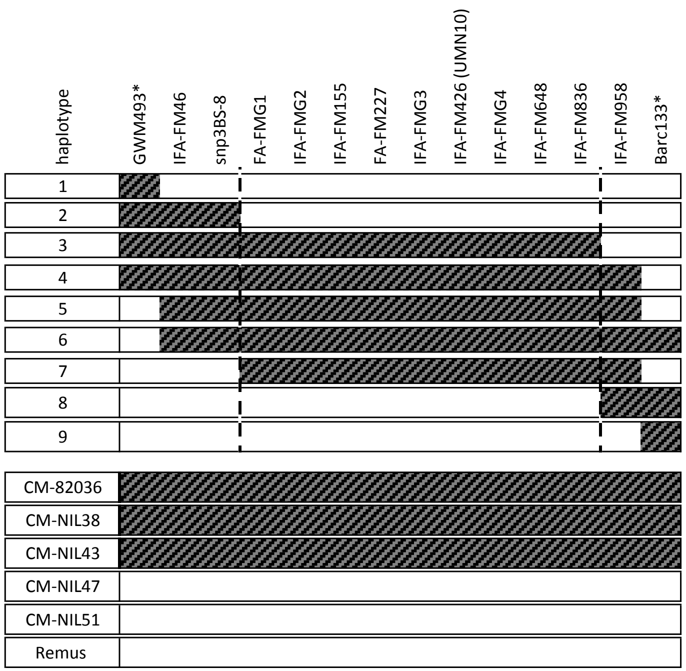

haplotype	FHB severity: lines / haplotype	FHB severity: number of inoculated heads	**FHB severity (mean number of diseased spikelets/head)**	FHB severity: standard deviation	FHB severity: **Resistance class (R/S)**	DON severity: lines / haplotype	DON severity: number of treated heads	**DON severity (mean number of DON-bleached spikelets/head)**	DON severity: standard deviation	DON severity: **Resistance class (R/S)**
1	32	458	**5.2[ac]**	±0.1	**S**	9	181	**9.6[a]**	±2.1	**S**
2	3	197	**3.6[ab]**	±0.1	**R/S**	3	82	**10.0[a]**	±2.4	**S**
3	3	71	**1.9[b]**	±0.2	**R**	3	52	**0.2[b]**	±0.4	**R**
4	17	364	**2.1[b]**	±0.1	**R**	5	96	**0.1[b]**	±0.2	**R**
5	1	10	**2.0[b]**	±0.4	**R**					
6	24	262	**2.2[b]**	±0.1	**R**	5	108	**0.2[b]**	±0.2	**R**
7	1	11	**1.9[b]**	±0.4	**R**	1	38	**0[b]**	±0	**R**
8	3	116	**4.4[a]**	±0.1	**S**	3	50	**10.2[a]**	±2.1	**S**
9	16	321	**7.2[c]**	±0.1	**S**	6	125	**10.3[a]**	±1.4	**S**
CM-82036		37	**1.9[b]**	±0.2	**R**		21	**0.4[b]**	±0.1	**R**
CM-NIL38		53	**2.0[b]**	±0.2	**R**		19	**0.6[b]**	±0.3	**R**
CM-NIL43		56	**2.2[b]**	±0.2	**R**		17	**1.0[b]**	±0.1	**R**
CM-NIL47		53	**4.7[a]**	±0.3	**S**		21	**9.2[a]**	±1.1	**S**
CM-NIL51		49	**6.2[ac]**	±0.2	**S**		15	**9.9[a]**	±0.6	**S**
Remus		45	**18.8[d]**	±0.2	**S**		20	**10.4[a]**	±2.5	**S**

Graphical illustration of the nine haplotypes and the control lines in the Fhb1 interval for 16 markers and their phenotypes for Fusarium and DON resistance. Resistant (CM-82036) alleles for the markers are illustrated by dashed boxes, whereas susceptible (Remus) alleles are shown in white. The FHB/DON resistance levels are measured in number of diseased/DON-bleached spikelets per head 26 days after Fusarium inoculation or DON infiltration. The dotted lines define the interval harboring Fhb1. *R*, resistant and *S*, susceptible. Haplotypes significantly different from each other are depicted by different letters (FHB severity: mean $LSD_{\alpha=5\%} = 2.0$, DON severity: mean $LSD_{\alpha=5\%} = 3.0$)

Supplementary File Fig. S5. Phenotype after DON infiltration of CM-NIL38 (left) and CM-NIL51 (right).

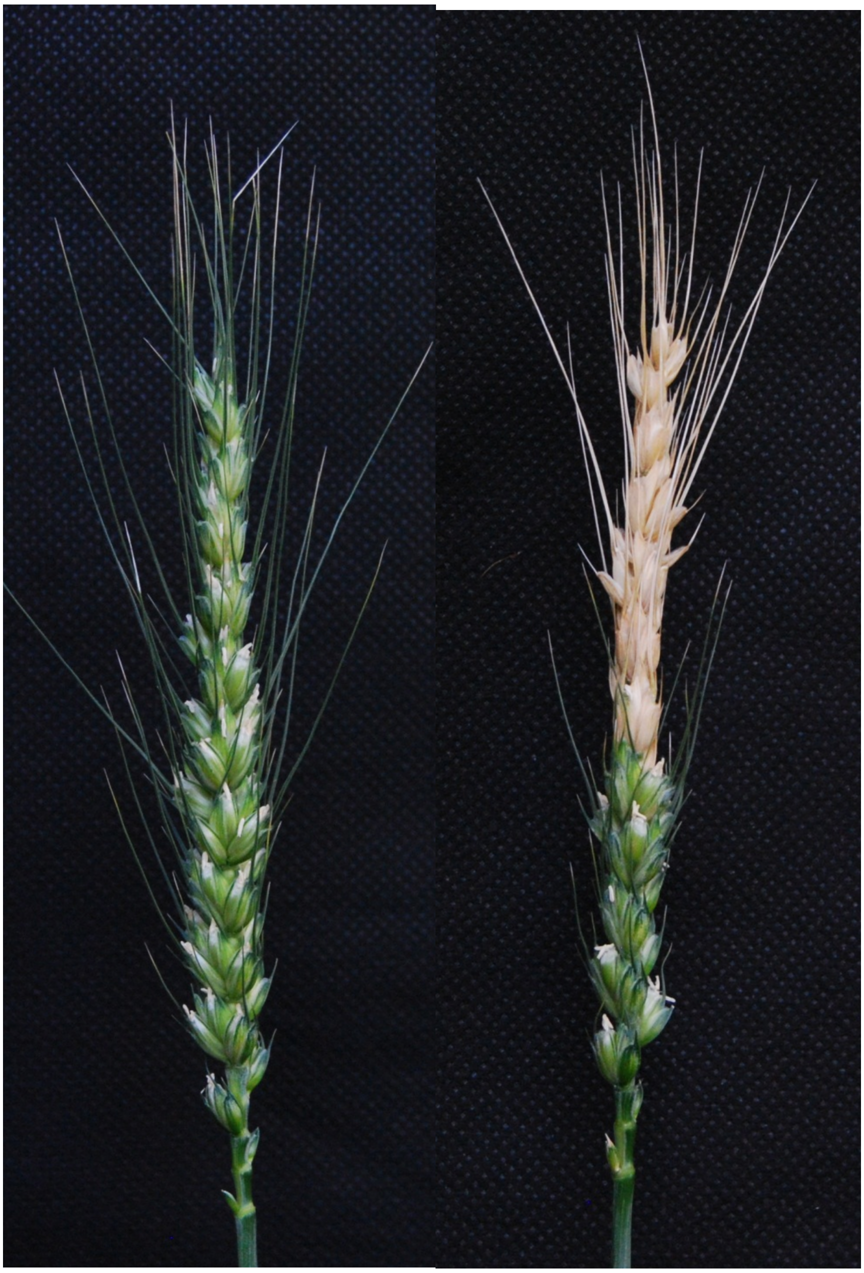

Supplementary File Fig. S6. Amino acid alignment of TAA_ctg0954b.00390.1 and gene #20 both encoding a putative sarcoplasmic reticulum histidine-rich calcium-binding protein .

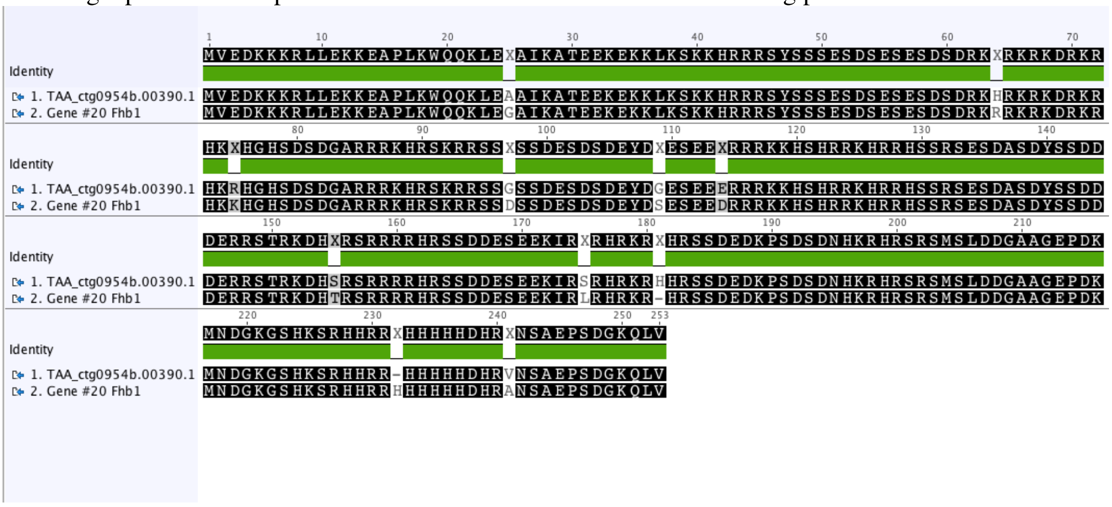

Supplementary File Fig. S7. Amino acid alignment of the *Fhb1* encodedd NB-ARC domain containing protein and XP_010230626 encoded in the susceptible Chinese Spring.

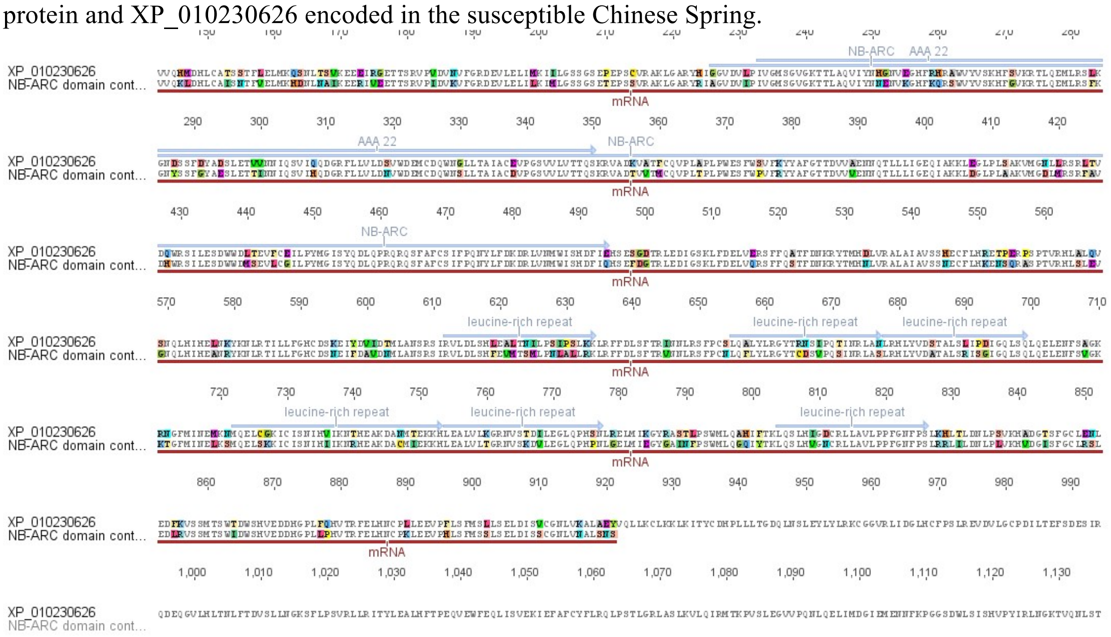

Supplementary File Fig. S8. Amino acid aligment of a Fhb1 encoded alanyl tRNA synthase (1. Alanyl tRNA Synthase CM) and the corresponding gene on the susceptible cv. Chinese Spring (Alanyl tRNA Synthase CS).

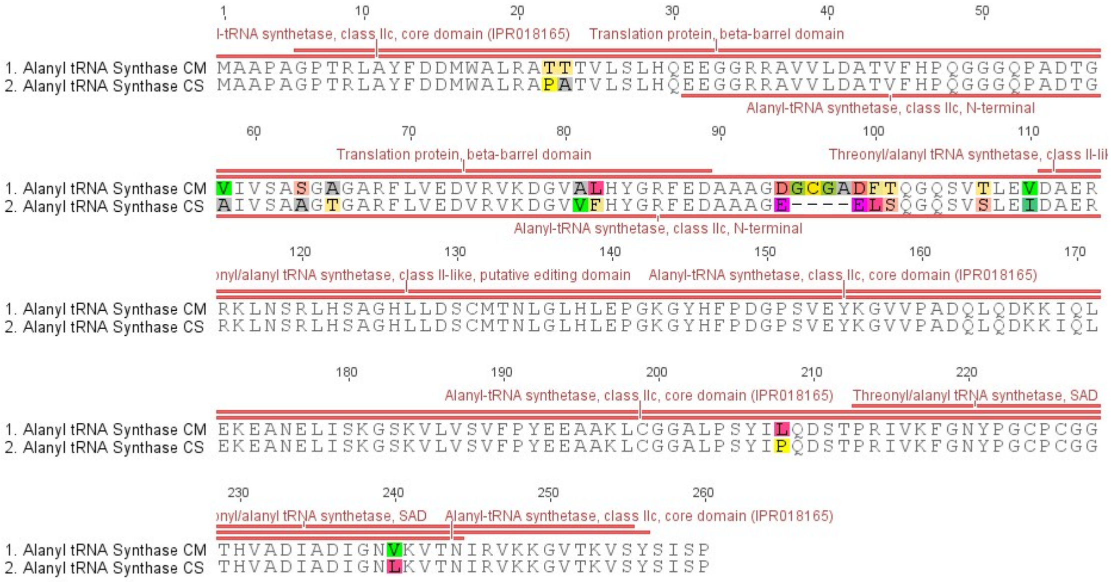

Supplement: Supplementary file 1 — Supplementary material 1 (PDF 3321 kb) [file 122_2016_2727_MOESM1_ESM.pdf]
